# The characterization of human oral mucosal fibroblasts and their use as feeder cells in cultivated epithelial sheets

**DOI:** 10.4155/fsoa-2017-0074

**Published:** 2017-09-06

**Authors:** Kazunari Higa, Yoshiyuki Satake, Jun Shimazaki

**Affiliations:** 1 Department of Ophthalmology/Cornea Center, Tokyo Dental College Ichikawa General Hospital, 5-11-13 Sugano, Ichikawa, Chiba 272-8513, Japan

**Keywords:** differentiation, epithelial sheet, oral mucosa fibroblast

## Abstract

**Aim:**

To characterize human oral mucosa middle interstitial tissue fibroblasts (hOMFs) and their application in the cultivation of epithelial sheets.

**Methodology:**

hOMFs were cultured with methylcellulose to form cell clusters. hOMFs amplified in adhesive culture were analyzed by flow cytometry, and were found to differentiate into multiple cell types suitable for the cultivation of human corneal epithelial sheets. hOMFs were expanded from clusters to analyze CD56 and PDGFRα expression.

**Results:**

These cells showed similar differentiation patterns as keratocytes, and similar expression patterns as mesenchymal and neural cells. Furthermore, we established human corneal epithelial sheets using hOMFs.

**Conclusion:**

hOMFs may be of neural crest origin and possess multipotent differentiation capacity, and are suitable for use as an autologous cell source for corneal regeneration.

## Lay abstract

Mesenchymal stem cells can be found in the middle interstitial tissue just under the human oral mucosa epithelium. To mediate the application of these mucosal stem cells, called human oral mucosa middle interstitial tissue fibroblasts (hOMFs), in a clinical setting, their characteristics were analyzed to determine whether they can differentiate into corneal stroma cells called keratocytes and develop human corneal limbal epithelial sheets by coculture with hOMFs as feeder cells as a substitute for 3T3 feeder cells from mice. hOMFs show potential as a new cellular source for autologous regeneration of mesenchymal or neural crest-derived tissues.

Mesenchymal stem cells (MSCs) have multilineage differentiation potential for mesodermal and neural lineages [1–3]. Neural crest origin is a key characteristic of potent stem cells from the dermal papillae, epidermal bulge area of the hair follicles, dental pulp and corneal stroma [4–8]. Oral mucosal tissue is developmentally surrounded by the jawbone and masseter tissues from the neural crest-origin pharyngeal arches, and it also originates from the embryonic neural crest [9]. Several recent studies have reported that oral mucosa-derived stem cells contain neural crest-origin cells that potentially differentiate into neurons [10,11]. We also reported that human oral mucosal nonepithelial stromal cell populations are capable of multiple types of differentiation in a manner similar to hMSCs, which contain cells of neural crest origin [12]. Fibroblasts from human oral mucosa middle interstitial tissue (hOMFs) may be a potential cell source for autologous regeneration of neural crest-derived tissues such as osteocartilaginous cephalic tissues, peripheral nerves, oral hard tissues and corneal stroma.

Oral mucosa epithelial cells have been used as an autologous cell source for corneal epithelium reconstruction in bilateral ocular surface diseases to take advantage of bioengineering-cultivated epithelial cell sheets for transplantation with or without substrates such as temperature-sensitive dishes, fibrin and amniotic membranes [13–16]. Sourcing transplant cells from the oral mucosa also offers the advantages of quick healing and minimal invasiveness, and is a simple procedure that causes only slight discomfort for patients. Recently, hOMFs have been shown to support the expansion of epithelial cells as an alternative to murine 3T3 feeder fibroblasts [17]. To mediate the application of more sophisticated hOMFs in a clinical setting, we isolated hOMFs using the methylcellulose culture method, analyzed their characteristics, investigated whether hOMFs differentiate into corneal stroma keratocytes, and developed human corneal limbal epithelial sheets by coculturing with hOMFs as feeder cells in place of murine 3T3 feeder cells.

## Materials & methods

### Tissue preparation & cell isolation

Oral mucosa from ten human patients was excised using an 8-mm punch biopsy tool (KAI Industries Co., Ltd., Gifu, Japan) under local anesthetic (xylocaine), following the approved protocol of the Tokyo Dental College Ethics Committee and with informed consent. The oral mucosa was separated into oral mucosa epithelium and oral mucosa nonepithelial tissues by treating with 2.4 U/ml dispase II (Roche, Mannheim, Germany) at 37°C for 1 h to digest the basement membrane, as described in previous studies [15,18]. Next, oral mucosa nonepithelial tissues were treated with 2 mg/ml collagenase (Wako Pure Chemical Industries, Ltd., Osaka, Japan) at 37°C overnight to isolate the hOMFs from these tissues [12]. hOMFs were cultured with Advance-Dulbecco's modified Eagle's medium (DMEM) containing 10% fetal calf serum (FCS) at 37°C in humidified air with 5% CO_2_ for 2 weeks.

### Generation of hOMF clusters

**Figure 1.**
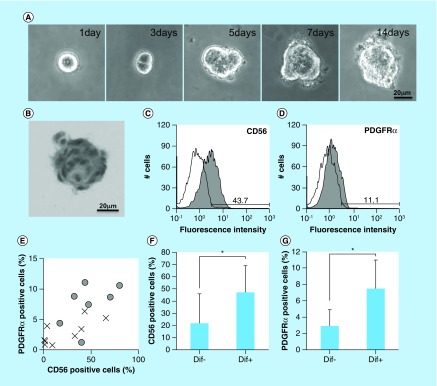
**Isolation of human oral mucosa middle interstitial tissue fibroblasts and analysis of differentiation.** **(A)** Phase-contrast micrographs showing temporal changes in clonal human oral mucosa middle interstitial tissue fibroblast growth in 0.8% methylcellulose culture for 2 weeks. Scale bar: 20 μm. **(B)** Hematoxylin and eosin staining on day 14 of **(A)**. Scale bar: 20 μm. **(C & D)** Flow cytometry analysis of expression of CD56 **(C)** and PDGFRα **(D)**. White histogram: isotype control; gray histogram: marker expression. Cell counts (number of cells) and fluorescence intensity are indicated on the ordinate and abscissa, respectively. **(E)** Association between differentiation and the expression of CD56 and PDGFRα. Ratios of CD56- and PDGFRα-positive cells are indicated on the ordinate and abscissa, respectively. Dots indicate each clone with (circles) or without (crosses) of one or more differentiated lineages. **(F & G)** Ratio of CD56- **(F)** or PDGFRα- **(G)** positive cells with (Dif+) or without (Dif-) differentiated lineage. Ratio of positive cells is indicated on the ordinate. Error bars and asterisks indicate SD (Dif+, n = 7; Dif-, n = 9) and p-values (CD56, p = 0.048; PDGFRα, p = 0.005), respectively.

To isolate human oral mucosa mesenchymal cells, we cultured the amplified hOMFs (8.0 × 10^3^ cells/ml was the concentration used to minimize cell-to-cell aggregation [19]) with 0.8% methylcellulose in Advance-DMEM containing 10% FCS on low-adhesive plates (HydroCell, CellSeed, Tokyo, Japan) to avoid the attachment of cells to the bottom of the plates. After 2 weeks at 37°C in humidified air with 5% CO_2_, cells formed clusters (Figure 1). The clusters were placed on adhesive plates, and the cells were amplified by explant adhesive culture. After several passages, we used a total of 16 clonally grown cells for the following experiments.

### Flow cytometry

Cells from passages 2–3 were harvested, washed twice with phosphate-buffered saline (PBS), incubated for 10 min with 2 ml TrypLE express (Invitrogen, CA, USA) at 37°C and then collected. The cells were fixed with 4% paraformaldehyde (PFA; Wako Pure Chemical Industries, Ltd.) at 4°C for 5 min, washed with 2 ml 0.1% sodium azide (Wako Pure Chemical Industries, Ltd.) and centrifuged at 3500 r.p.m. for 5 min. Cell suspensions of 1.0 × 10^6^ cells/ml were incubated with individual antibodies (Table 1) for 30 min on ice and measured with a flow cytometer (EPICS XL; Beckman Coulter, CA, USA). Flow cytometry data from 5000 events were analyzed using FlowJo software (Tree Star Inc., OR, USA). In the control samples, isotype antibodies conjugated with fluorescein isothiocyanate or phycoerythrin (Beckman Coulter) were used.

**Table 1.** **Antibodies for flow cytometric analysis.**

	**Antibody**		**Clone**	**Isotype**	**Company**
Adhesion molecules	CD31	Platelet endothelial cell adhesion molecule-1	JC70A	Ms-IgG1	Dako Cytomation

	CD44	Hyaluronate receptor	2C5	Ms-IgG2a	R&D Systems, Inc.

	CD56	NCAM	N901[NKH-1]	Ms-IgG1	IOTest

	CD146	MCAM	128018	Ms-IgG1	R&D Systems, Inc.

	CD166	Alcam	105902	Ms-IgG1	R&D Systems, Inc.

Growth factors and cytokine receptors	CD105	Endoglin	166707	Ms-IgG1	R&D Systems, Inc.

	PDGFRα	Platelet-derived growth factor receptor alpha polypeptide, mesenchymal cell mitogen receptor	PRa292	Ms-IgG1	R&D Systems, Inc.

Hematopoietic markers	CD14	LPS receptor	RMO52	Ms-IgG2a	IOTest

	CD34	Hematopoietec stem cell marker	563	Ms-IgG1	BD

	CD45	Leukocyte antigen	2D1	Ms-IgG1	R&D Systems, Inc.

Others	CD73	5′-Terminal nucleotidase	AD2	Ms-IgG1	BD

	CD90	Thy-1	Rhy-1A1	Ms-IgG2a	R&D Systems, Inc.

	STRO-1	Bone marrow stromal cells, erythroid precursors and dental pulp	Stro-1	Ms-IgGM	R&D Systems, Inc.

	FSP-1	Fibroblast specific protein-1 (S100A4)	–	Rabbit IgG	Dako Cytomation

LPS: Lipopolysaccharide; MCAM: Melanoma cell adhesion molecule; NCAM: Neural cell adhesion molecule.

### *In vitro* differentiation

Upon reaching semiconfluency, the cells were washed in PBS and incubated with TrypLE (Invitrogen) at 37°C for 7 min. The collected cells were seeded at a density of 1.0 × 10^4^ cells/cm^2^ in 4-well chamber slides (LAB-TEK; Nalge Nunc International, NY, USA) and 5.0 × 10^3^ cells/cm^2^ in 6-well plates (MA, USA). For chondrogenesis using pellet cultures, 5.0 × 10^5^ cells were placed in 15-ml polypropylene tubes (BD Falcon, NJ, USA) and pelletized at 440 × *g* for 5 min at 4°C.

For osteogenic induction, cells were cultured in Advance-DMEM containing 10% FCS. Once they reached confluency, the cultures were further grown in osteogenic induction medium (Lonza, MD, USA) containing dexamethasone, ascorbate, mesenchymal cell growth supplement (MCGS), L-glutamine, β-glycerophosphate and gentamycin (GA-1000; Lonza). The medium was changed three-times per week, and cultures were analyzed after 2 weeks.

For adipogenic induction, cells were cultured in Advance-DMEM containing 10% FCS until they reached confluency. The cultures were further grown in adipogenic induction medium (Lonza) containing human recombinant insulin, L-glutamine, MCGS, dexamethasone, indomethacin, 3-isobutyl-methylxanthine and GA-1000 (Lonza). The control groups were grown in adipogenic maintenance medium (Lonza) containing human recombinant insulin, L-glutamine, MCGS and GA-1000 (Lonza). The medium was changed three-times a week, and cultures were analyzed after 2 weeks.

For chondrogenic induction, cells were cultured in Advance-DMEM containing 10% FCS until they reached confluency. The cultures were further grown in complete chondrogenic induction medium (Lonza) containing dexamethasone, ascorbate, insulin–transferrin–selenium (Lonza) supplement, GA-1000, sodium pyruvate, proline, L-glutamine and TGF-β3 (Lonza). The control groups were grown in incomplete chondrogenic induction medium without TGF-β3. The medium was changed three-times a week and cultures were analyzed after 2 weeks.

For neurogenic induction, cells were cultured on fibronectin- (PromoCell, Heidelberg, Germany) coated chambers or plates in Advance-DMEM containing 10% FCS until they reached confluency. Chambers or plates were coated with 10 μg/ml fibronectin for 10 min at room temperature. The cultures were further grown in neurogenic differentiation medium (PromoCell). The control groups were grown in Advance-DMEM containing 10% FCS. The medium was changed three-times a week, and cultures were analyzed after 2 weeks.

For keratocyte induction, hOMFs were cocultured with human limbal epithelial cells and keratocytes isolated from donor corneas obtained from the Northwest Eye Bank following corneal transplantation. Limbal rims of corneoscleral tissue were prepared by careful removal of excess sclera, iris and corneal endothelium. Limbal epithelial cells were isolated as previously described [20]. Dispersed epithelial cells were seeded onto inserts with coculture medium. After endothelium transplantation, donor corneal stroma buttons were treated with 2 mg/ml collagenase (Wako Pure Chemical Industries, Ltd.) at 37°C overnight to isolate the keratocytes from these tissues. Cocultures (1.0 × 10^5^ cells/well) were grown in Advance-DMEM containing 10 ng/ml recombinant human EGF and 10 ng/ml bFGF for 2 weeks using Transwell culture inserts (Costar Corning, NY, USA). The medium was changed three-times a week.

### Alkaline phosphatase staining

After culturing for 2 weeks, cells were fixed in 0.4% cold PFA, rinsed twice with alkaline phosphatase (ALP) solution (100 mM Tris-HCl, pH 9.5, 100 mM NaCl, 10 mM MgCl_2_) and stained with a bromocresol purple solution (Roche) for 30 min at 37°C. Slides were rinsed with deionized water and observed under a microscope.

### Alizarin red S staining

Cultured cells were fixed in 70% cold ethanol for 10 min and rinsed with deionized water. The fixed cells were stained with alizarin red S solution (Sigma-Aldrich, MO, USA) for 10 min at room temperature. Stained cells were rinsed with deionized water and observed under a microscope.

### Oil red O staining

The cells cultured in chambers were fixed in 4% cold PFA for 10 min and rinsed with 60% isopropyl alcohol (Wako Pure Chemical Industries, Ltd.). Oil Red O stain (200 mg; Sigma-Aldrich) was dissolved in 10 ml 60% isopropyl alcohol and filtered. Fixed cells were stained with 2% Oil Red O solution for 5 min at room temperature. Slides were then rinsed with deionized water, counterstained with hematoxylin (Wako Pure Chemical Industries, Ltd.) for 15 min and observed under a microscope.

### Safranin O stain

The induced micromasses were frozen in Tissue-Tek OCT Compound (Sakura Finetek, Tokyo, Japan), and sliced into 5-μm-thick sections. The sections were fixed in 10% formalin solution (Wako Pure Chemical Industries, Ltd.) for 10 min. The sections were rinsed with deionized water and stained with 6% Safranin O solution (Chroma, Munster, Germany) for 5 min at room temperature. The sections were then rinsed with deionized water, counterstained with hematoxylin for 15 min and observed under a microscope.

### Immunostaining

Frozen sections or chamber slides were fixed for 10 min in 2% PFA (Wako Pure Chemical Industries, Ltd.) or acetone (Wako Pure Chemical Industries, Ltd.) on ice. Frozen sections and slides were blocked by incubation with 3% normal donkey serum (Chemicon Int., Inc., CA, USA), 1% bovine serum albumin (Sigma-Aldrich) and 0.3% Triton X-100 (Sigma-Aldrich) for permeabilization of cell membranes for 1 h at room temperature. Antibodies (Table 2) were applied for 90 min at room temperature, followed by incubation with fluorescein isothiocyanate-, rhodamine- and/or Cy3-conjugated secondary antibodies. Primary antibodies were substituted with isotype antibodies as negative controls (Table 2). After three washes with PBS, the sections or slides were incubated with 0.5 μg/ml 4′,6-diamidino-2-phenylindole (Dojindo Laboratories, Tokyo, Japan) at room temperature for 5 min. Finally, sections were washed three-times in PBS and coverslipped using an aqueous mounting medium containing an antifading agent (Fluoromount/Plus; Diagnostic Biosystems, CA, USA). Images were observed under a florescence microscope (Axioplan2 Imaging; Carl Zeiss, Inc., NY, USA).

**Table 2.** **Antibodies for immunostaining and Western blot.**

**Antibody**		**Clone**	**Isotype**	**Company**
Collagen type II	Fibril-forming interstitial collagen, articular cartilage marker	II-4C11	Ms-IgG1	MP Biomedicals

NFM	Neurofilament heavy chain, neuron-specific protein	NF-09	Ms-IgG2a	Abcam

βIII-tubulin	Tubulin b-4, neuron-specific marker	TuJ-1	Ms-IgG2a	R&D Systems, Inc.

Keratocan	Small leucin-rich proteoglycan, expressed cornea stroma	–	Goat IgG	SantaCruz

Keratin 3	Corneal epithelium differentiation marker and palate epithelium marker	AE5	Ms-IgG1	PROGEN

Keratin 12	Corneal epithelium differentiation marker	–	Rabbit IgG	TransGenic, Inc.

Collagen Type IV	Basement membrane collagen, basement menbrane component of corneal limbal epithelial cells	–	Goat IgG	Southern Biotec

Laminin	Extracelluar matrix, basement menbrane component of corneal epithelial cells	NU-01-LA3	Ms-IgG1	Cosmo Bio

Occludin	Tight junctions protein for barrier function of both epithelial and endothelial cells	OC-3F-10	Ms-IgG1	Invitrogen

ZO-1	Tight junctions-associated proteins link between the cytoskeleton and the tight junction	ZO1-1A12	Ms-IgG1	Invitrogen

Ki67	Nuclear protein expressed during all active phases of the cell cycle (G1, S, G2, M)	MIB-1	Ms-IgG1	Dako Cytomation

Ms-IgG1	Negative control mouse IgG1	DAK-GO1		Dako Cytomation

Ms-IgG2a	Negative control mouse IgG2a	DAK-GO5		Dako Cytomation

Goat IgG	Normal goat IgG	–		SantaCruz

Rabbit IgG	Normal rabbit IgG	–		Dako Cytomation

### Reverse transcription polymerase chain reaction & real-time polymerase chain reaction analysis

Total RNA was isolated from cultured cells using the SV Total RNA Isolation System (Promega, WI, USA) according to the manufacturer's recommendations. Complementary DNA (cDNA) was prepared from total RNA with 0.25 M dithiothreitol, 5× reaction buffer, RNase inhibitor and avian myeloblastosis virus reverse transcriptase (Takara Bio, Inc., Shiga, Japan) by incubating a 25-μl mixture at 41°C for 1 h. This cDNA was used as a template for polymerase chain reaction (PCR) amplification. Amplifications (1 μl cDNA in a total reaction volume of 50 μl) were run for three cycles of 95°C for 1 s, 52°C for 30 s and 72°C for 20 s, then 25 cycles of 95°C for 30 s, 52°C for 30 s and 72°C for 20 s on a GeneAmp PCR System 9700 thermocycler (Applied Biosystems, CA, USA). Primer sequences, reaction conditions and the size of each product are listed in Table 3. The amplification of glyceraldehyde-3-phosphate dehydogenase (GAPDH) was performed in the same manner to check cDNA quality. Amplification products were separated by electrophoresis on 1.5% agarose gels (Takara Bio, Inc.).

**Table 3.** **Primer sequences and product size of reverse transcription polymerase chain reaction.**

**Primer**		**Sequence (5′→3′)**	**Product size (bp)**
ALP	Alkaline phosphatase, hydrolase enzyme	GGCGGCAGACTTTGGTTT	552

		CCCGTGGCAACTCTATCTT	

OPN	Osteopontin, bone sialoprotein 1	TGGCTAAACCCTGACCCATCTC	548

		TAACTGTCCTTCCCACGGCTGT	

PPAR-γ2	Peroxisome proliferator-acticated receptor-gamma2, adipocyte-specific transcription factor	GGTCAGCGGGAAGGACTTTA	516

		GATCCAGTGGTTGCAGATTA	

Leptin	Adipose-derived hormones	GCCAGAGTTCCTTCCCTTAA	508

		CAAGCTGTGCCCATCCAAAA	

Aggrecan	Chondroitin sulfate proteoglycan 1	TCCTGGAAGCTCTTCTCAGT	510

		ATGCCCAAGACTACCAGTGG	

BMP-6	Bone morphogenetic protein-6, chondrogenic marker	CTCGGGGTTCATAAGGTGAA	451

		ACCGCATAACATGGGGCTTC	

Nestin	Neural stem cell marker, type VI intermediate filament	GCGTTGGAACAGAGGTTGGAG	385

		GCACAGGTGTCTCAAGGGTAG	

NFH	Neurofilament heavy chain, neuron-specific protein	TGAACACAGACGCTATGCGCTCAG	398

		CACCTTTATGTGAGTGGACACAGAG	

βIII-tubulin	Tubulin β-4, neuron-specific marker	GTCTTACGAGGAGCTGCAGACGCA	233

		CCACATAGCTGCAGCTTGCCATCT	

ALDH3A1	Aldehyde dehydrogenase 3 family member A1	ACTCAGCAGGACGAGCTCTAC	495

		GGGTCACAGAGGATGTAGTC	

Lumican	SLRP family/Class II subfamily of proteins	CCACAACAACCTGACAGAGTGT	488

		CAAGTTGATTGACCTCCAGG	

Keratocan	Cornea-specific KSPG	CCTCCAAGATTACCAGCCAA	367

		TTCCATCCAGACGGAGGTAG	

Keratin 3	Corneal epithelium differentiation marker and palate epithelium marker	GACAATAATCGTTCCCTGG	434

		TTGCGGTAGGTGGCGATCT	

Keratin12	Corneal epithelium differetiation marker	CAACGACATGAGGGCGCA	481

		AATTGACTGTGCTTGAGA	

Keratin14	Stratified squamous epithelial marker	ACTACCTGCAGCCGCCAGTT	1417

		CAGTTCTTGGTGCGAAGGAC	

Collagen type IV	Collagen type IV, basement membrane component	ATACCTGGAAGGGCAAGAGAA	484

		TGGCACCGGCTGATTTTCTGG	

Laminin	Extracellular matrix protein	TGCGAGCATGTCAGGATTTC	961

		TCAGTTCCAACTCTCCCATTG	

GAPDH	Glyceraidehyde-3-phosphate dehydrogenase, internal control	ACCACAGTCCATGCCATCAC	452

		TCCACCACCCTGTTGCTGTA	

KSPG: Keratan sulfate proteoglycan; SLRP: Small leucine-rich proteoglycan.

Real-time PCR was carried out using an ABI PRISM 7500 Sequence Detection System (Applied Biosystems). Probes, primers and custom-formatted TaqMan array plates were purchased from Applied Biosystems. Reactions were performed according to manufacturer's methods and run in triplicate in three independent experiments. 18S rRNA was used as an internal control to normalize the variability in expression levels. Comparative expression ratios were calculated with the ΔΔCt method, using the following formula:
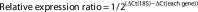



The values are expressed as the control group (hMSCs) normalized to 1.

### Cultivation of human corneal epithelial sheets

Bolheal fibrin sealant was purchased from Teijin Pharma LTD (Tokyo, Japan), and constituted as previously reported [21]. In brief, a solution containing 40 mg of human fibrinogen and 0.18 U of thrombin was diluted in 7.5 ml saline, and 0.3 ml was spread rapidly onto the upper chambers of 6-well Transwell culture inserts (Costar Corning). After 2 h, polymerized fibrin-coated upper chambers were obtained and stored at 4°C. Human corneas were obtained from eye banks in the USA for investigational purposes. Limbal rims of corneoscleral tissue were prepared by careful removal of excess sclera, iris and corneal endothelium. Epithelial sheets were isolated as previously described [20]. Dispersed epithelial cells (2 × 10^5^ cells/ml) were seeded onto fibrin-coated wells with supplemental hormonal epithelial medium [22] containing 666 KIU/ml aprotinin (Wako Pure Chemical Industries, Ltd.), and cocultured with mytomycin C (MMC)-treated 3T3 fibroblasts (Figure 6A). The culture was submerged in medium until confluence, cultured in air–liquid interface for 4 days and finally incubated without aprotinin for 4 days. The transparency of the epithelial sheets with culture inserts (Figure 1C) and after removing culture inserts (Figure 1D) was assessed by comparing the visibility of the character ‘F’.

**Figure 2.**
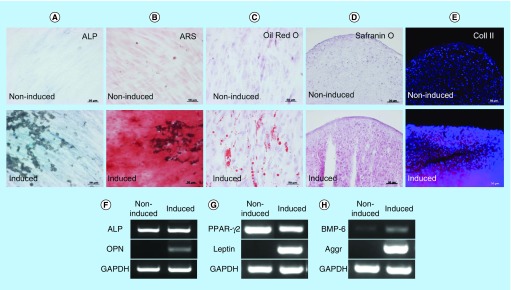
**Induction of differentiation into mesenchymal cells.** **(A)** ALP staining, **(B)** ARS staining, **(C)** oil red O staining, **(D)** safranin O staining and **(E)** immunohistochemistry of Coll II in noninduced (upper panel) and induced (lower panel) human oral mucosa middle interstitial tissue fibroblasts. Scale bars: 50 μm. Reverse transcription-PCR analysis shows **(F)** osteogenic (ALP, OPN), **(G)** adipogenic (PPAR-γ, leptin) and **(H)** chondrogenic (BMP-6, aggrecan)-specific gene expression. Primers used are listed in Table 3. GAPDH was used as an internal control. Left lane: noninduced human oral mucosa middle interstitial tissue fibroblasts; right lane: induced human oral mucosa middle interstitial tissue fibroblasts. ALP: Alkaline phosphatase; ARS: Alizarin red S; Aggr: Aggrecan; Coll II: Collagen type II; GAPDH: Glyceraldehyde-3-phosphate dehydogenase; OPN: Osteopontin; PPAR-γ: Peroxisome proliferator-activated receptor-γ.

**Figure 3.**
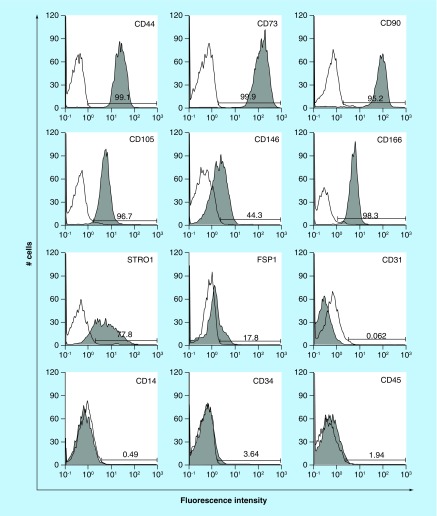
**Expression of characteristic human oral mucosa middle interstitial tissue fibroblasts markers by flow cytometry.** Antibodies for flow cytometric analysis are listed in Table 1. White histogram: isotype control; gray histogram: marker expression indicated at the upper right of each panel. Cell counts (number of cells) and fluorescence intensity are indicated on the ordinate and abscissa, respectively. Numbers in panels indicate the positive rate.

**Figure 4.**
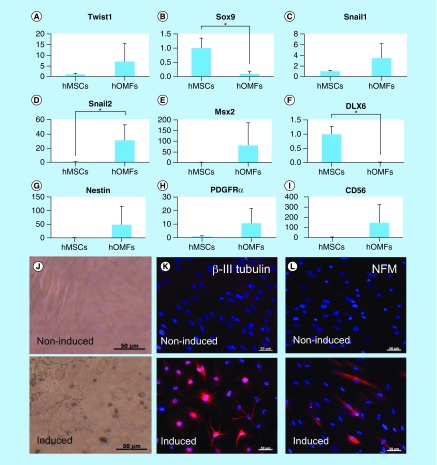
**Expression of early neural crest (Twist1, Sox9, Snail1 and Snail2), ectomesenchymal (MSX2 and DLX6) and neural markers.** **(A–I)** Real-time PCR of early neural crest (Twist1, Sox9, Snail1, Snail2, CD56 and PDGFRα), ectomesenchymal (MSX2 and DLX6) and neural stem cell (nestin) markers. *Y*-axis indicates relative expression compared with the control group levels (hMSCs), which were normalized to 1. Error bars indicate SD (n = 4). **(J)** Phase-contrast images of noninduced (upper panel) and induced (lower) hOMFs. **(K & L)** Immunocytochemical staining of βIII-tubulin **(K)** and NFM **(L)**. Noninduced (upper panel) and induced (lower) hOMFs. Nuclei were stained with 4′,6-Diamidino-2-phenylindole (DAPI). Scale bars: 50 μm. hMSC: Human mesenchymal stem cell; hOMF: Human oral mucosa middle interstitial tissue fibroblast; NFM: Neurofilament medium.

**Figure 5.**
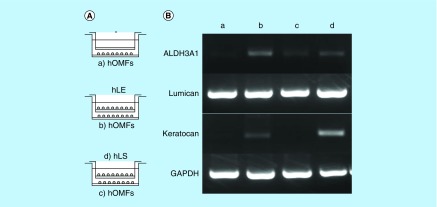
**Differentiation of human oral mucosa middle interstitial tissue fibroblasts into cells with a keratocyte phenotype.** **(A)** Illustrated cultivation methods of hOMFs with hLE cells and hLS cells. **(B)** Comparison of keratocyte phenotype in each cell type by reverse transcription PCR. GAPDH was used as an internal control. **(a)** hOMFs alone. **(b)** hOMFs cocultured with hLE. **(c)** hOMFs cocultured with hLS. **(d)** hLS cocultured with hOMFs. GAPDH: Glyceraldehyde-3-phosphate dehydogenase; hLE: Human limbal epithelial cell; hLS: Human limbal stromal cell; hOMF: Human oral mucosa middle interstitial tissue fibroblast.

**Figure 6.**
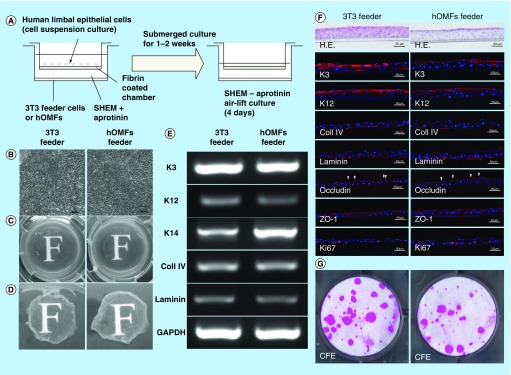
**Cultivation of human limbal epithelial sheets with two types of feeder cells.** **(A)** Illustrated cultivation methods of human limbal epithelial sheets. **(B)** Phase-contrast micrographs of epithelial sheets with 3T3 (left panel) and hOMF (right panel) feeder cells. Scale bars: 50 μm. **(C)** Photographs of both types of human limbal epithelial sheets. **(D)** Macroscopic view of both types of human limbal epithelial sheets after removing culture inserts. **(E)** Comparison of both types of human limbal epithelial sheet phenotypes by reverse transcription polymerase chain reaction. GAPDH was used as an internal control. **(F)** Histochemical comparison of the phenotypes of both types of human limbal epithelial sheets. Arrowheads indicate occludin expression. Scale bars: 50 μm. **(G)** Comparison of CFE in each sheet. Colonies were stained with rhodamine B after 2 weeks. CFE: Colony-forming efficiency; GAPDH: Glyceraldehyde-3-phosphate dehydogenase; H.E.: Hematoxylin & eosin; hOMF: Human oral mucosa middle interstitial tissue fibroblast; SHEM: Supplemental hormonal epithelial medium.

### Colony-forming efficiency

To evaluate the proliferative potential of cells in the cultured sheets, MMC-treated 3T3 fibroblasts were used in a colony-forming efficiency (CFE) assay as previously described [23–25]. NIH 3T3 fibroblasts in DMEM containing 10% FCS were treated with MMC (4 μg/ml) for 2 h at 37°C and then treated with trypsin-EDTA and plated at a density of 6 × 10^5^ cells with supplemental hormonal epithelial medium (5% FCS) in 34-mm culture dishes. Single cells were prepared from epithelial cell sheets treated with Acutase (Innovative Cell Technologies, Inc., CA, USA) for 60 min at 37°C. Each dish was seeded at 3 × 10^2^ cells/dish. Growth capacity was evaluated on day 14, when cultured cells were stained with rhodamine B (Wako Pure Chemical Industries, Ltd.) for 30 min.

### Statistical analyses

Significant differences between groups were determined by Student's *t*-test (Excel; Microsoft, WA, USA). p < 0.05 was considered statistically significant.

## Results

### Selection & characterization of clusters

A key characteristic of immature mesenchymal cells from the dermal papillae, epidermal bulge area of the hair follicles, dental pulp and corneal stroma is their neural crest origin [4–8]. To select clusters containing immature mesenchymal cells expressing neural crest-related molecules, we performed flow cytometry to detect CD56 and PDGFRα expression in hOMFs expanded from 16 clusters (Figure 1A–D). hOMFs containing relatively high ratios of CD56- and PDGFRα-positive cells were shown to be of similar differentiation status (Dif+) to osteoblasts and/or adipocytes or chondrocytes (Figure 1E–G). In differentiated hOMFs with the highest ratio of PDGFRα-positive cells, induced cells showed positive staining for ALP, alizarin red S, oil red O, safranin O and collagen type II (Figure 2A–E). Reverse transcription polymerase chain reaction (RT-PCR) results showed that induced cells also expressed the expected differentiation markers, such as ALP and osteopontin (osteogenic), peroxisome proliferator-acticated receptor-γ2 and leptin (adipogenic) and BMP-6 and aggrecan (chondrogenic; Figure 2F–H). To reveal additional characteristics of hOMFs, we performed expression analysis of antigens with MSCs by flow cytometry. hOMFs showed higher expression of CD44, CD73, CD90, CD105, CD146, CD166 and STRO-1; and lower expression of fibroblast-specific protein, CD31 (vascular endothelial cells), CD14, CD34 and CD45 (myelopoietic cells; Figure 3). To compare these cells with commercially available bone marrow-derived MSCs, we analyzed the expression of neural crest- and neuron-related genes by real-time PCR. In hOMFs, Snail2 expression levels were significantly higher and Sox9 and DLX6 expression levels significantly lower than those in MSCs (Figure 4A–F). Although the expression levels of neural-related genes were not significantly different between hOMFs and MSCs, these genes trended toward higher expression in hOMFs (Figure 4G–I). Furthermore, in neural differentiation, hOMFs displayed neurite-like structures and expressed neural markers (βIII-tubulin and neurofilament medium; Figure 4J–L).

### Keratocyte differentiation

To induce corneal stromal keratocytes from hOMFs, hOMFs were cocultured with human corneal stroma-derived cells (Figure 5A). hOMFs showed similar expression profiles of ALDH3A1, lumican and keratocan to keratocytes by RT-PCR (Figure 5B).

### Application of hOMFs in place of 3T3 feeder cells in human corneal limbal epithelial sheets

To examine whether hOMFs are suitable feeder cells for human corneal epithelial sheets, we engineered epithelial sheets with hOMF feeder cells and compared them to 3T3 feeder cells using human limbal epithelial sheet cultivation methods (Figure 6A). Epithelial sheets using hOMFs as feeder cells showed a similar cobblestone appearance to cells cultivated in epithelial sheets with 3T3 feeder cells (Figure 6B). To observe the transparency of these epithelial sheets, a critical factor in ocular surface diseases involving poor or lost visual acuity, such as chemical burns, Stevens–Johnson syndrome and Sjogren's syndrome, we compared the transparency of the epithelial sheets by assessing the visibility of the character ‘F.’ Epithelial sheets using hOMFs were of similar transparency to epithelial sheets with 3T3 feeder cells (Figure 6C & D). In reconstruction of the corneal epithelium by sheet transplantation, it is critical that the transplants are similar to the corneal phenotype. Therefore, the sheets must show maintenance of the corneal phenotype. To examine the phenotype of epithelial sheets using hOMF feeder cells, we compared both types of epithelial sheets using RT-PCR and immunohistochemistry. Epithelial sheets using hOMF feeder cells produced uniform cell layers that expressed the differentiation markers K3 and K12, similar to sheets with 3T3 feeder cells (Figure 6E–G). Epithelial sheets using hOMFs also expressed the progenitor marker K14 (Figure 6E). Basement membrane components (collagen type IV and laminin) and tight junction-related proteins (occludin and ZO-1) were also expressed in the basal layer and superficial layer of stratified epithelial sheets, respectively (Figure 6F). To estimate the proliferative potency of the epithelial sheets, we assessed the expression of proliferation markers and CFE in both types of epithelial sheets. Epithelial sheets using hOMFs as feeder cells also expressed the proliferation marker Ki67, as assessed by immunohistochemistry (Figure 6F). The CFE was similar (3T3: 10.3 ± 0.3%; hOMFs: 9.2 ± 0.7%; n = 3) in cells dissociated from both types of sheets (Figure 6G).

## Discussion

In this study, we showed the differentiation capacity of hOMFs, with a significantly high ratio of CD56- and PDGFRα-positive cells (Figure 1F & G). CD56-positive cells have increased clonogenic and proliferative potential and a unique chondrocyte and pancreatic differentiation capacity [26]. PDGFRα is also related to the high growth capacity and potency of MSCs isolated from bone marrow [27]. These data suggested that CD56 and PDGFRα expression might affect the differentiation capacity or MSC potency of hOMFs. MSCs preferentially expressing STRO-1, PDGFRα and TWIST-1 have high growth capacity [27]. These data were similar to our data (Figures 2D, 3 & 4A). Moreover, CD56 and PDGFRα are the most important markers of neural crest origin, as they play major roles in the migratory properties of cells [28,29]. Neural crest-derived stem cells differentiated into mesenchymal or neuronal lineages in human oral mucosa lamella propria and continued to express neural crest and neural markers [10]. We previously reported that neural crest and neural markers were expressed in hOMFs, which might contain both MSCs and neural crest-origin cells [12]. In this study, hOMFs were isolated as clusters expanded from a single cell or a small number of cells using the methylcellulose culture method, and clusters were selected based on expression of the neural crest-related molecules CD56 and PDGFRα. To culture well-established epithelial sheets, we had to prepare stable and well-characterized hOMF feeder cells. We showed that the hOMFs were consistent with previous data and displayed more clonality, the ability to differentiate into multiple cell types including osteoblasts, adipocytes or chondrocytes, and expressed MSC markers (Figures 2 & 3). Davies *et al*. demonstrated that clonally derived oral mucosa progenitor cells are multipotent and capable of generating both mesenchymal and neuronal cell lineages [11]. Although they used a different isolation method from our methylcellulose culture method, the results were similar. Therefore, it is likely that the isolated hOMFs were neural crest-origin progenitor cells.

Cornea-specific keratocans are involved in a phenotypic change in activated keratocytes, which are neural crest-derived corneal stromal cells [30,31]. Recent studies reported corneal stromal differentiation of MSCs derived from bone marrow, adipose tissue and the umbilical cord [32–34]. In this study, we detected keratocan expression when hOMFs were cocultured in the presence of corneal limbal epithelial cells (Figure 5). hOMFs also expressed various transcription factor genes including Twist1, Snail1, Snail2 and Msx2 (Figure 4), which are involved in the development of the neural crest and in neural crest cell differentiation [1,35–41]. This suggests that keratocan expression and neural crest elements in hOMFs play an important role in the differentiation and maintenance of the keratocyte phenotype.

Cultivation of stratified epithelial cell sheets requires coculturing with feeder cells, as keratocytes interact with corneal epithelial cells. The currently preferred cultivation method requires the use of xenobiotic 3T3 feeder cells from mouse embryonic fibroblasts in the culture system. Avoiding xeno-derived materials decreases the risk of unknown infections; recent studies have reported the production of stratified human corneal and oral mucosal epithelial sheets by coculturing with human MSCs, human dermal fibroblasts or hOMFs as substitutes for murine 3T3 feeder cells [17,42–45]. O'Callaghan *et al*. reported that hOMFs are a more suitable alternative to 3T3 feeder cells than human limbal fibroblasts in the production of epithelial sheets [17]. For clinical use, we are required to supply stable and well-characterized epithelial sheets and information on the preparation of the feeder cells. Feeder cells exhibit different characteristics based on their tissue of isolation, harvest site and the individual donors. Therefore, we isolated more clonal hOMFs using the methylcellulose culture method and selected clusters based on expression of the neural crest-related molecules, CD56 and PDGFRα. We also developed stratified human corneal epithelial sheets with hOMFs as feeder cells in this study (Figure 6). Factors important for ocular surface reconstruction include low-affinity neurotrophin receptor p75, an early neural crest-related factor and receptor of NGF, brain-derived neurotorophic factor and neurotrophin-3/4/5, expressed in human corneal epithelium, oral mucosa epithelium, skin and neurons as regulatory factors that mediate cell survival, differentiation, proliferation and plasticity [46–49]. Recent studies have reported that the limbal stem/progenitor cells were preserved by culture conditions combining EGF and NGF [50], and human limbal epithelial cells on amniotic membranes were expanded through NGF signaling [51]. We found that immature cells were preserved in human limbal epithelial sheets cocultured with hOMF feeder cells, because these sheets expressed K14 and were able to form epithelial cell colonies (Figure 6E–G). These findings suggest that corneal limbal epithelial sheets were supported by the preservation, proliferation and differentiation of limbal stem/progenitor cells by hOMF feeder cells containing neural crest-derived cells.

## Conclusion

In this study, we isolated and cultured hOMFs containing neural crest-origin cells, and demonstrated their ability to differentiate into both mesenchymal and neural cell lineages, including corneal keratocytes, and their utility as a substitute for xenobiotic 3T3 feeder cells in corneal or oral mucosal-stratified epithelial cell sheets. Although further characterization of hOMFs is necessary, hOMFs are a potential cellular source for autologous regeneration of mesenchymal or neural crest-derived tissues.

## Future perspective

Although mesenchymal cells from bone marrow and adipose tissue are currently applied in clinical settings, there are no reports on the use of hOMFs. Mesenchymal cells are considered to have different characteristics depending on the tissue from which they are harvested. By identifying the characteristics and abilities of hOMFs containing neural crest-derived cells, we anticipate their use as a new autologous cell source in refractory diseases of neural crest-derived tissues such as ocular surfaces.

**Summary points**

**Background**

- Human oral mucosa middle interstitial tissue fibroblasts (hOMFs) may be a potential cellular source for autologous regeneration of neural crest-derived tissues.
- To mediate the application of hOMFs in a clinical setting, hOMFs were isolated and analyzed, and human corneal limbal epithelial sheets were developed by coculturing with hOMFs as feeder cells substituting for murine 3T3 feeder cells.

**Materials & methods**

- hOMFs were cultured with methylcellulose, forming cluster cells.
- To select clusters containing immature mesenchymal cells, we performed flow cytometry to detect CD56 and PDGFRα expression in hOMFs.
- Amplified hOMFs were analyzed by flow cytometry and examined to differentiate cell types.
- To determine the suitability of hOMFs as feeder cells in place of murine 3T3 feeder cells, human corneal epithelial sheets were cocultured with hOMFs and analyzed with reverse transcription polymerase chain reaction and immunohistochemistry.

**Results**

- hOMFs were selected based on CD56 and PDGFRα expression, indicating similarity to mesenchymal stem cells, and showed multilineage differentiation, including differentiation into corneal keratocytes.
- We successfully developed stratified human corneal epithelial sheets substituting hOMFs for murine 3T3 feeder cells.

**Discussion**

- Our findings suggest that isolated hOMFs were neural crest-origin progenitor cells.
- Our findings suggest that corneal limbal epithelial sheets were supported by the preservation, proliferation and differentiation of limbal stem/progenitor cells by hOMF feeder cells.
